# Study on Quality Characteristic of Chebulae Fructus and Its Adulterants and Degradation Pathway of Hydrolyzable Tannins

**DOI:** 10.3390/molecules29102399

**Published:** 2024-05-20

**Authors:** Jian Xu, Xiangdong Wang, Huijuan Yu, Xin Chai, Min Zhang, Hong-Hua Wu, Yuefei Wang

**Affiliations:** 1 National Key Laboratory of Chinese Medicine Modernization, State Key Laboratory of Component-Based Chinese Medicine, Tianjin Key Laboratory of TCM Chemistry and Analysis, Tianjin University of Traditional Chinese Medicine, Tianjin 301617, China; xjwyq123456@163.com (J.X.); xiangdongblue@163.com (X.W.); huijuanyu@tjutcm.edu.cn (H.Y.); chaix0622@tjutcm.edu.cn (X.C.); 2 Haihe Laboratory of Modern Chinese Medicine, Tianjin 301617, China

**Keywords:** Chebulae Fructus, hydrolyzable tannins, phenolcarboxylic acids, hierarchical cluster analysis, principal component analysis, quality analysis

## Abstract

Chebulae Fructus (CF) is known as one of the richest sources of hydrolyzable tannins (HTs). In this study, ultra-performance liquid chromatography coupled with a photodiode array detector method was established for simultaneous determination of the 12 common phenolcarboxylic and tannic constituents (PTCs). Using this method, quantitative analysis was accomplished in CF and other four adulterants, including Terminaliae Belliricae Fructus, Phyllanthi Fructus, Chebulae Fructus Immaturus, and Canarii Fructus. Based on a quantitative analysis of the focused compounds, discrimination of CF and other four adulterants was successfully accomplished by hierarchical cluster analysis and principal component analysis. Additionally, the total contents of the 12 compounds that we focused on in this study were unveiled as 148.86 mg/g, 96.14 mg/g, and 18.64 mg/g in exocarp, mesocarp, and endocarp and seed of CF, respectively, and PTCs were witnessed to be the most abundant in the exocarp of CF. Noticeably, the HTs (chebulagic acid, chebulanin acid, chebulinic acid, and punicalagin) were observed to be ultimately degraded to chebulic acid, gallic acid, and ellagic acid during sunlight-drying of the fresh fruits. As a result, our study indicated that CF and its adulterants could be distinguished by the observed 12 PTCs, which were mainly distributed in the exocarp of the fruits. The HTs were prone to degrade into the three simple phenolcarboxylic acids during drying or processing, allowing us to obtain a more comprehensive understanding of the PTCs, with great significance in the improved quality of CF and related products.

## 1. Introduction

Chebulae Fructus (CF) is the dried ripe fruit of *Termianlia chebula* Retz. or its variant *Terminalia chebula* Retz. var. *tomentella* Kurt [1], which is an umbrella medium-sized deciduous tree from the Combretaceae family which is widely distributed in China, India, Myanmar, and other Asian countries [2]. CF is recognized as the “king of medicine” in Tibetan medicine and a top-listed herb in Ayurvedic Materia Medica [3,4], possessing extraordinary bioactivities, such as antioxidant [5], hepatoprotective [6], neuroprotective [7], antimicrobial [8], and anti-diabetic activities [9]. CF’s health benefits rely on its intrinsic diverse phytochemicals, as exemplified by polyphenols including tannins [10], phenolcarboxylic acids, terpenoids, and flavonoids [11], among which the dominant constituent is hydrolyzable tannins (HTs). As is known, the fruit of *Terminalia chebula* is both edible and medicinal, which has been widely used in conventional folk medicine for a long time [9]. CF is frequently prescribed in many traditional herbal preparations, such as Sanzi powder in Mongolian medicine, Jiebai Wan in Tibetan medicine [1], and Triphala in Indian Ayurvedic medicine [12].

However, there were four other adulterants, including Chebulae Fructus Immaturus (CFI), Terminaliae Belliricae Fructus (TBF) [13], Phyllanthi Fructus (PF) [14], and Canarii Fructus (CAF) [15], which are frequently misused, mixed, and adulterated in clinical settings due to their similar names, appearance, and pharmacological effects. CFI, as the immature CF, is a popular folk medicine prescribed for a sore throat, pharyngitis, and dysentery [16]. Notably, CF, TBF, and PF are widely recognized as the polyherbal medicine “Triphala” [12], and both TBF and PF contain the similar major constituents to CF, including tannins, flavonoids, and triterpenoids. Thus, there is an urgent need for a global method of quality control for CF and its adulterants.

CF has been recognized as one of the richest sources of hydrolyzable tannins (HTs) [17]. There are three main types of HTs in CF [18,19], including (I) gallotannins, as represented by 1,3,6-tri-*O*-galloyl-β-d-glucose (TGG); (II) ellagitannins, as exemplified by corilagin (COR) and punicalagin (PUN); and (III) chebulic ellagitannins, such as chebulanin acid (CHI), chebulagic acid (CHG), and chebulinic acid (CHN). Notably, HTs are vulnerable to oxidation and hydrolysis before being degraded into phenolcarboxylic acids such as gallic acid (GA) and ellagic acid (EA) [20]. Nevertheless, whether and how the HTs degrade at high temperatures during drying and processing remains unclear. Yet, their distribution in different parts of the CF fruits is rarely touched upon.

At present, thin-layer chromatography (TLC) [21,22], high-performance liquid chromatography (HPLC) [2,23], and liquid chromatography-mass spectrometry (LC-MS) [24,25] have been applied for qualitative and quantitative analysis of the main PTCs in CF, CFI, and TBF. However, few studies have focused on the global determination of the PTCs constituents in CF, CFI, TBF, PF, and CAF, let alone the analytical method for discriminating CF from other adulterants.

Under this circumstance, herein, an ultra-high-performance liquid chromatography-photodiode array (UPLC-PDA) method was established for quantitative determination of the twelve major constituents, including six phenolcarboxylic acids [CA, GA, EA, urolithin M5 (UM5), 4-*O*-galloyl-(–)-shikimic acid (4GS), and 3-*O*-galloyl-(–)-shikimic acid (3GS)] and six HTs (PUN, COR, CHG, CHN, TGG, and CHI) in CF and its four adulterants. By the established method, the potential for discrimination was then evaluated by hierarchical cluster analysis and principal component analysis. Further, the distribution of these PTCs in different parts of CF and the degradation of the HTs during sunlight-drying were investigated. And finally, the degradation pathways of the representative HTs (CHG, CHI, CHN, and PUN) were proposed. Our study is dedicated to laying the foundation for a quality analysis of CF-related Chinese medicines and its more reasonable application of CF and related products.

## 2. Results and Discussion

### 2.1. Optimization and Methodological Validation of the Quantitative Analysis for the Focused Compounds

Traditional extraction methods, such as percolation and reflux, always require a long extraction time with insufficient or unsatisfactory extraction yields [26]. Ultrasonic extraction has been widely applied in the extraction of active ingredients from Chinese medicines, possessing unique advantages such as shorter extraction time, higher extraction rate, and lower extraction temperature. Herein, by taking the instability of HTs into account, ultrasonic extraction coupled to UPLC-PDA was applied to determine the observed 12 PTCs in CF, CFI, TBF, PF, and CAF.

**Figure 1:**
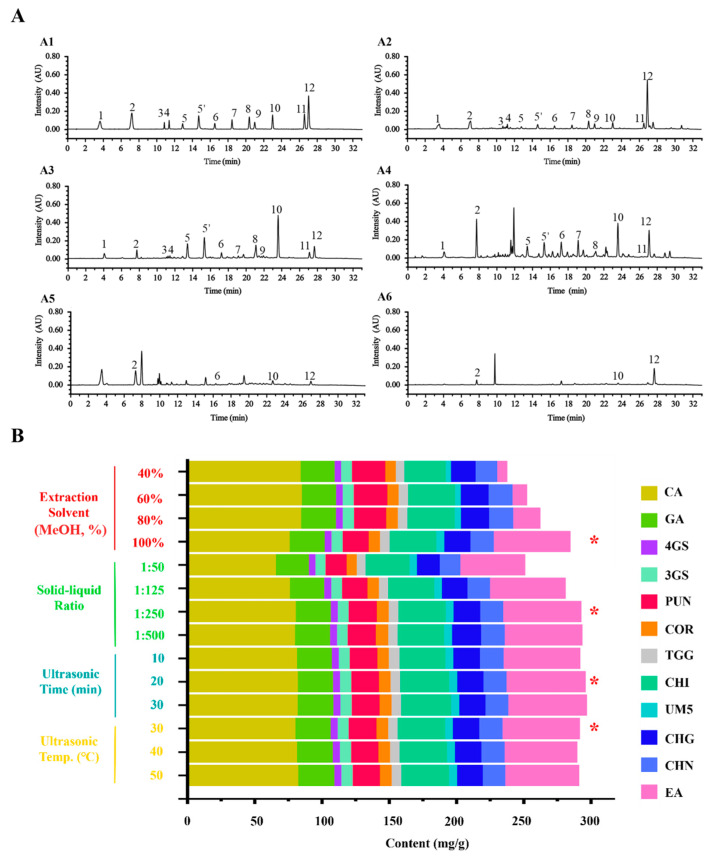
UPLC chromatograms of the mixed reference solution and the tested sample solutions (**A**) and the histogram for the contents of 12 observed constituents under different extraction conditions (**B**). (**A**): UPLC chromatogram of the mixed reference solution (**A1**) and UPLC chromatograms of the tested sample solutions of CF (**A2**), CFI (**A3**), TBF (**A4**), PF (**A5**), and CAF (**A6**); (**B**): CA (1), GA (2), 4GS (3), 3GS (4), PUN (5 and 5′), COR (6), TGG (7), CHI (8), UM5 (9), CHG (10), CHN (11), and EA (12). *, the optimal extraction solvent, solid-liquid ratio, ultrasonic time, and ultrasonic temperature.

The representative chromatograms of mixed standard solution (A1) and the samples solution (A2–A6) are displayed in Figure 1. Moreover, the factors that may affect the extraction outcome of the tested compounds were investigated and optimized, including the extraction solvent, solid–liquid ratio, ultrasonic time, and ultrasonic temperature, as presented in Figure 1B. The extraction solvent exhibited a significant impact on the extraction yields of the observed constituents. The results of methodological validation for the simultaneous quantification of twelve observed compounds in CF are shown in Table 1. The calibration curves of twelve compounds were established with the determination coefficient (*r*^2^) exceeding 0.9998, allowing the strong linear correlation within the tested ranges. The limit of detection (LOD) and limit of quantification (LOQ) were 0.0998–0.9779 μg/mL and 0.3992–1.956 μg/mL, respectively. For these quantified compounds, the relative standard deviations (RSDs) of intra-day and inter-day precisions were below 2.7% and 1.0%, respectively. The RSDs of repeatability were below 3.0%, and the stability test showed that the determined compounds in the sample solution remained stable for 12 h, with RSDs below 1.2%. The mean recovery ranged from 97.41% to 113.4%, with RSDs below 6.5%. Thus, the ultrasonic extraction coupled with UPLC-PDA quantitative method was successfully established with favorable applicability in our study.

### 2.2. Quantitative Determination of the Twelve Observed Constituents in CF, CFI, TBF, PF, and CAF

Tannins are polyphenolic compounds that are universally distributed in the plant kingdom, and are accumulated extensively in foods and Chinese medicines, performing a variety of pharmacological activities [27]. Herein, the contents of twelve constituents in CF and other adulterants were determined, as shown in Supplementary Figure S1. Among the 12 constituents determined in both CF and CFI, 9 constituents (except 4GS, 3GS, and UM5) could be tested in TBF, 4 of them (GA, COR, CHG, and EA) could be observed in PF, while 3 of them (GA, CHG, and EA) could be detected in CAF. Notably, CFI, as the immature fruit of CF, differs from CF with great disparity in terms of its contents of PUN, CHG, and CHN. The content fluctuations of the observed compounds in 35 batches of CF indicated the batch-to-batch inconsistency in CF samples. Specifically, the contents of PUN (6.90–81.35 mg/g; RSD, 70.01%), CHG (6.82–156.84 mg/g; RSD, 74.36%), and CHN (3.48–182.65 mg/g; RSD, 99.13%) in CF varied considerably among different batches, presumably arising from the different places of production (India, Myanmar, and some provinces in China) or the different processing methods. And as one of the major components of CF, the content of HTs was as high as 369.81 mg/g, accounting for 83.82% of the total contents of all the determined compounds.

Chemometrics methods, including hierarchical clustering analysis (HCA) and principal component analysis (PCA), were conducted to evaluate the discrimination potential for CF and four adulterants by the quantitative result.

**Figure 2:**
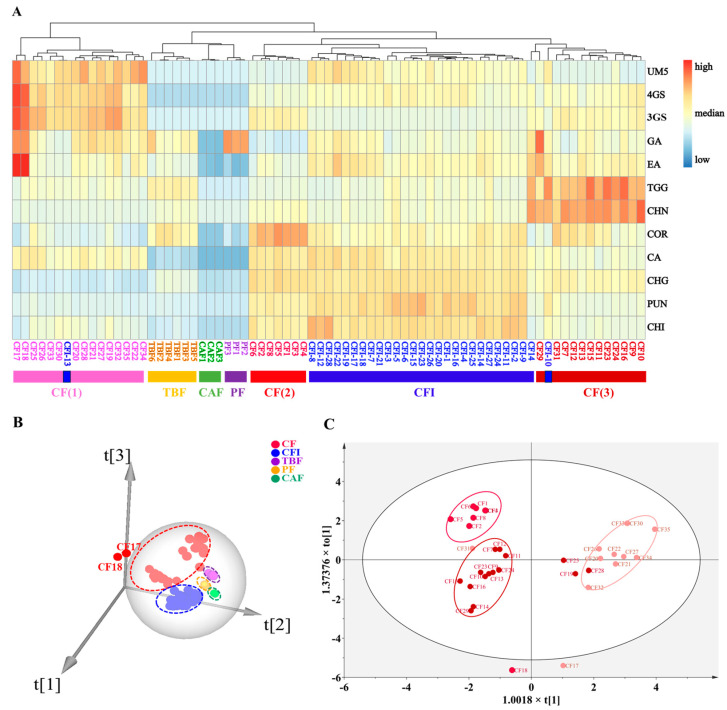
Clustering heatmap analysis (**A**) based on the 12 observed constituents’ contents in CF (35 batches), CFI (28 batches), TBF (6 batches), PF (3 batches), and CAF (3 batches), discrimination of CF, CFI, TBF, PF, and CAF by PCA (**B**), and discrimination of the whole fruit and the flesh of CF by OPLS-DA (**C**).

A clustering heatmap (Figure 2A) was created based on HCA to show the relative contents of PTCs in the clustered samples. As shown, CF was satisfactorily distinguished from the other four Chinese medicines (CFI, TBF, PF, and CAF) except for CFI-10 and CFI-13 samples. Furthermore, the inconsistency of the CF samples was witnessed based on the HCA clustering, and CF samples were divided into three groups: group CF(1) with high contents of phenolcarboxylic acids (UM5, 4GS, 3GS, GA, and EA), group CF(2) with high contents of HTs (COR, CHG, PUN and CHI), and group CF(3) with relatively high contents of other HTs (TGG and CHN).

Before comprehensive PCA analysis, CF, CFI, TBF, PF, and CAF samples were separately pre-analyzed by PCA to remove the outliers, as shown in Supplementary Figure S2. Then, as displayed, samples CFI-13 and CFI-22 with relatively higher levels of GA and EA were removed as outliers before further analysis, presumably due to the massive hydrolysis of HTs in CFI. As shown in Figure 2B, CF, CFI except for CFI-13 and CFI-22, TBF, PF, and CAF were further successfully discriminated by the first principal component (PC1), second principal component (PC2), and third principal component (PC3), accounting for 34.6%, 29.6%, and 18.8%, respectively. The good fit of the PCA model was displayed by R^2^X (0.952) and goodness of prediction (Q^2^ = 0.781). As has been determined, the higher R^2^X value (close to 1) shows the better suitability of the model, whereas the higher Q*^2^* value (greater than 0.5) indicates the stronger predictivity of the developed model [28]. Moreover, as shown in Supplementary Figure S3, CF, CFI, and TBF were clustered closely into the center of the sphere, while PF and CAF are close to the edge of the sphere, illustrating that these Chinese medicines could be distinguished from each other by the PCA model. However, samples CF17 and CF18 were located outside of the sphere to indicate their higher contents of phenolcarboxylic acids (CF17, 137.52 mg/g; CF18, 141.24 mg/g) than other samples. The reason for this phenomenon may be the existence of massive hydrolysis of HTs triggered by the prolonged storage time or improper storage condition.

As mentioned above, the results of both HCA and PCA analyses prompted us to further discuss the cause of the inconsistency of CF samples by OPLS-DA analysis. As shown in Figure 2C, the clustered result of OPLS-DA analysis for CF was nearly consistent with that of HCA analysis. Interestingly, we found that a majority of whole fruits for the CF samples were located on the left side of the circle, and the flesh of CF samples fell on the right side of the circle. Meanwhile, the HTs content in whole fruit CF samples was significantly higher than that in the flesh of CF, indicating that preservation by whole fruits before processing and extraction was beneficial for retaining HTs. As shown in Supplementary Figure S4A, CHG, UM5, PUN, CHN, 3GS, and 4GS were filtered out by variable importance for the projection (VIP) value (VIP > 1.0), which were regarded as chemotaxonomic markers for discriminating flesh and whole fruit samples of CF. The results of the permutation tests indicate that the model has good predictive ability and can be applied to the discrimination study of CF (Supplementary Figure S4B,C).

### 2.3. Distribution of the Observed Constituents in Different Medicinal Parts of CF

Different parts of medicinal plants frequently display quite varied chemical compositions, which give rise to different therapeutic values [29]. For a long time, CF has been accepted as medicinal part of *Terminalia chebula*, benefiting from its high PTCs content. As we know, in practical therapeutic applications, CF is either prescribed as a whole fruit or in the form of flesh composed of exocarp and mesocarp. Therefore, the distribution of PTCs in various parts of CF was investigated in this study.

**Figure 3:**
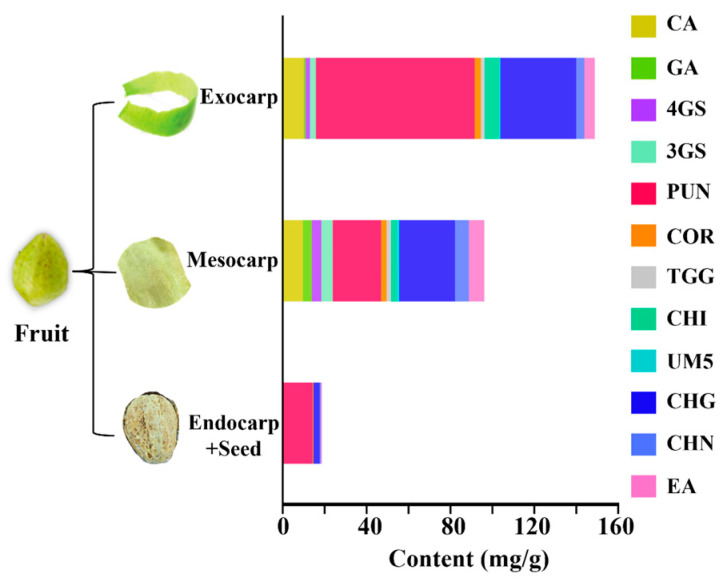
Histogram for the contents of the observed 12 constituents in different parts of CF.

As shown in Figure 3, the contents of PTCs in different parts of CF varied significantly with the total contents of the 12 studied compounds at 148.86 mg/g, 96.14 mg/g, and 18.64 mg/g in the exocarp, mesocarp, and endocarp and seeds of CF, respectively. The content of HTs in exocarp (127.41 mg/g) was significantly higher than that in mesocarp (63.67 mg/g), while the content of phenolcarboxylic acids in exocarp (21.44 mg/g) was lower than that in mesocarp (32.47 mg/g). The different level of HTs in different parts may be related to the number of tannin cells, which are responsible for synthesis, transport, accumulation, and distribution during the fruit development [30]. Notably, PUN and CHG were predominantly distributed in the exocarp, mesocarp, and endocarp and seed of CF. And the content of PUN in exocarp reached 75.69 mg/g, greatly exceeding that in mesocarp (23.07 mg/g) and endocarp and seeds (13.87 mg/g). Among the six HTs determined in CF, PUN was one of the ellagitannins that are frequently reported to possess antioxidant, hepatoprotective, anti-atherosclerotic, and antiproliferative activities against tumor cells [31].

As noticed, endocarp and seed contained trace amounts of PTCs, including CHG (2.99 mg/g), COR (0.37 mg/g), CHN (0.61 mg/g), 4GS (0.19 mg/g), 3GS (0.20 mg/g), and EA (0.42 mg/g). That is to say, the most of the PTCs needed for the pharmacological effects were allocated in the exocarp and mesocarp of CF, and endocarp and seeds could be removed from CF before being used in further medicinal applications concerning the PTCs. Additionally, the flesh of CF with the largest weight ratio of the whole CF fruit (above 50%) contains the highest total content of PTCs (GA, EA, COR, CHG, pentagalloyl glucoses, and casuarinins), supporting the clinical use of the flesh of CF [32]. For instance, in clinical research, the efficacy of Jiebai Wan pills made with the flesh of CF has been found to be obviously superior to that of the pills made with the whole CF fruits [33]. Furthermore, our previous study revealed that whole-fruit preservation before processing and extraction was beneficial for the conservation of the active tannic ingredients in CF, such as CHG and CHN [34]. Accordingly, we speculated that the tannic constituents undergo certain degradation during collection, preservation, and processing prior to being prescribed in a clinical setting.

### 2.4. Discovery on Degradation of the Observed Hydrolyzable Tannins during the Drying Process of CF

In this study, the content variations of PTCs in CF were observed during the sunlight-drying process to reveal the transformation pattern of HTs in CF. To ensure the accuracy of the experimental outcomes, we strictly controlled the weight of single fruit and employed the standard processing method documented in the Chinese Pharmacopoeia.

**Figure 4:**
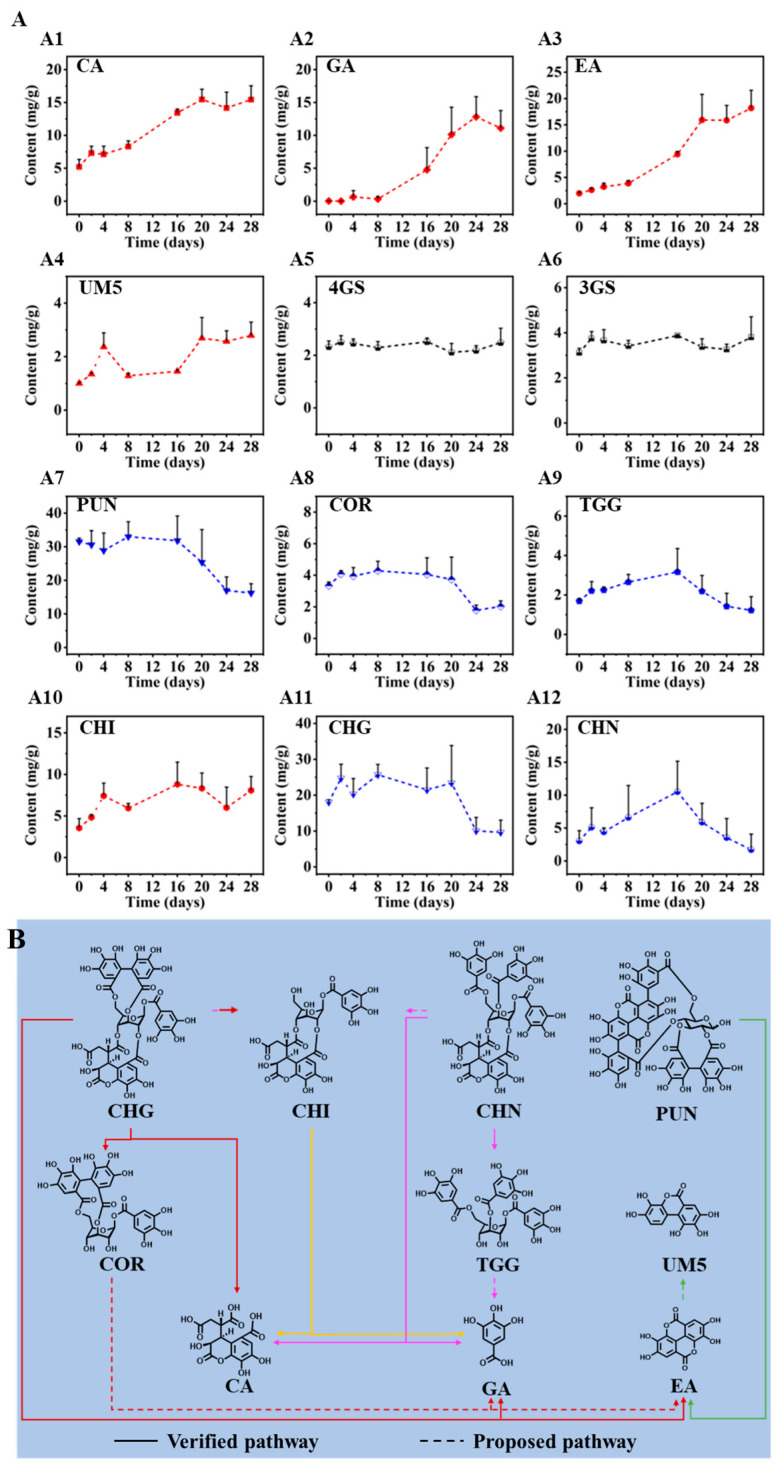
Dynamic changes in the contents of the observed 12 constituents over time (**A**) and the proposed degradation pathways (**B**) during the sunlight-drying of CF. CA (**A1**), GA (**A2**), EA (**A3**), UM5 (**A4**), 4GS (**A5**), 3GS (**A6**), PUN (**A7**), COR (**A8**), TGG (**A9**), CHI (**A10**), CHG (**A11**), CHN (**A12**). (**A**): The red, black, and blue lines indicate increased, steady, and decreased trends, respectively; (**B**): The arrows in red, yellow, purple, and green represents the degradation pathways from CHG, CHI, CHN, and PUN, respectively.

As shown in Figure 4(A1–A12), the contents of different PTCs in CF showed different trends during 28 days of sunlight-drying. For phenolcarboxylic acids, the contents of CA (A1), GA (A2), and EA (A3) significantly increased, UM5 (A4) slightly increased, while 4GS (A5) and 3GS (A6) were witnessed at steady levels. For HTs, the contents of PUN (A7), COR (A8), and CHG (A11) obviously decreased, the content of CHI (A10) increased, while the contents of TGG (A9) and CHN (A12) showed similar trends of initially increasing and subsequently decreasing. Accordingly, the degradation pathways of PTCs during sunlight-drying of CF were proposed as shown in Figure 4B. Specifically, CHG, CHI, and CHN may be degraded to afford large amounts of CA during the sunlight-drying process. COR, TGG, CHG, CHI, and CHN were prone to producing GA. And the hexahydroxydiphenoyl (HHDP) groups may be easily degraded from CHG, COR, and PUN to produce large amounts of EA that may be transformed into UM5. As reported, urolithins could be formed from EA by the initial loss of one of the two lactones and subsequent removal of the hydroxyl groups by intestinal microbiota, which were considered as the bioactive products obtained from EA or ellagitannins in vivo [35]. Meanwhile, it was reported that CHG can be degraded at high temperature to produce COR, CA, and EA [36]. CHN could be structurally degraded to produce TGG, which was further transformed into GA, resulting in an initial increase and then a subsequent decrease in TGG. And CHI may be easily produced by the loss of a HHDP moiety from CHG or the loss of galloyl groups from CHN.

### 2.5. Verification of the Degradation Pathways of Hydrolyzable Tannins from CF at High Temperature

**Figure 5:**
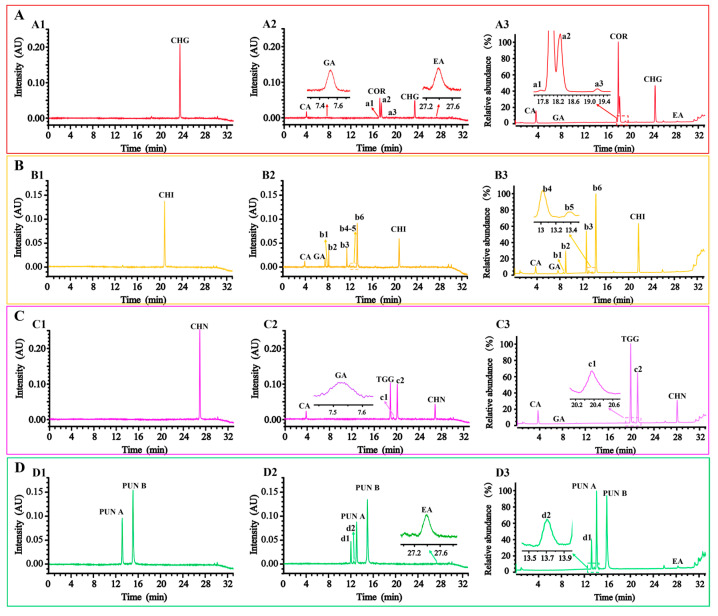
Representative UPLC chromatograms and total ion current chromatograms of CHG (**A**), CHI (**B**), CHN (**C**), and PUN (**D**) and their degraded products in the cultured samples under 60 °C for 24 h. (1) UPLC chromatograms of 0 h exposed samples [CHG (**A1**), CHI (**B1**), CHN (**C1**), and PUN (**D1**)], (2) UPLC chromatograms of 24 h exposed samples [CHG (**A2**), CHI (**B2**), CHN (**C2**), and PUN (**D2**)], and (3) total ion current chromatograms of 24 h exposed samples [CHG (**A3**), CHI (**B3**), CHN (**C3**), and PUN (**D3**)]. And, compounds a1–a3, b1–b6, c1–c2, and d1–d2 were the degradation products of CHG (**A**), CHI (**B**), CHN (**C**), and PUN (**D**), respectively.

To validate the influence of temperature on the degradation of the focused HTs, four HTs (CHG, CHI, CHN, and PUN) and their degradation products were qualitatively analyzed in the cultured samples at 60 °C for 24 h. As shown in Figure 5(A1–A3), two ester bonds at 2- and 4- positions of glucose in CHG were hydrolyzed to form COR, CA, and products a1–a3. The difference in the molecular weight was 18 Da between CHG (*m*/*z* 953.0936) and products a1–a3 (*m*/*z* 971.1026), which shared the same fragments of 633 Da and 337 Da, suggesting that they were produced by hydrolyzation of one of the ester bonds in the chebuloyl moiety of CHG (Supplementary Figure S5). And it can be deduced that products a1–a3 might be neochebulagic acid and its two isomers [37]. As shown in Figure 5(B1–B3), CHI was hydrolyzed to produce CA and GA as characteristic degraded constituents, as well as b1–b6. The fragment ions of product b2 (*m*/*z* 331.0691) were detected at *m*/*z* 169.0153 (GA), indicating that b2 was galloyl-substituted glucose, which was determined to be 1-*O*-galloylglucose by MS comparison and the standard compound (Supplementary Figure S6A). Products b1 and b3–b6 produced characteristic fragment ions of chebuloyl unit (*m*/*z* 337.0222). The quasi-molecular ion of b1 was 517.0857, speculating that b1 was generated by hydrolytic cleavage of the galloyl ester bond at the 1- position and one of the two chebuloyl ester bonds at the 2- or 4- position (Supplementary Figure S6B). Products b3–b6 (*m*/*z* 669.0961) were deduced to be phyllanemblinin E and phyllanemblinin F or their isomers [38] (Supplementary Figure S6C), with a quasi-molecular ion being 18 Da more than that of CHI, indicating that the ester bond at the 2- or 4- position of the glucose in CHI was hydrolyzed. Similarly, as shown in Figure 5(C1–C3), CHN was degraded to form CA, GA, TGG, and products c1 and c2. The difference between CHN (*m*/*z* 955.1085) and products c1–c2 (*m*/*z* 973.1186) is 18 Da (H_2_O), indicating that one of the ester bonds was hydrolyzed at the 2- or 4- position of glucose in CHN. Accordingly, products c1–c2 were inferred to be neochebulinic acid [18] and its isomers (Supplementary Figure S7). As shown in Figure 5(D1–D3), the HHDP unit of PUN could be easily hydrolyzed at high temperature to form EA. Meanwhile, the two newly generated products d1 and d2 were produced and preliminarily witnessed at *m*/*z* 1083.0615 ([M–H]^−^), 1065.0519 ([M–H–H_2_O]^−^), and 721.0333 ([M–H–C_16_H_10_O_10_]^−^) in MS spectra, as two isomers of PUN (Supplementary Figure S8).

In summary, the common chebulic ellagitannins, gallotannins, and ellagitannins in CF could be easily degraded to produce CA, GA, and EA by hydrolyzation of ester bonds during the drying process, resulting in a decrease in HTs and an increase in CA, GA, and EA. The transformation and degradation of tannic constituents are always reported, which satisfactorily clarifies the fluctuation of contents for the detected compounds and variation in the biological activities of the tested sample.

## 3. Materials and Methods

### 3.1. Reagents and Materials

Methanol was purchased from Sigma-Aldrich Inc. (St. Louis, MO, USA). Formic acid was bought from Shanghai Aladdin Bio-Chem Technology Co., Ltd. (Shanghai, China). Dimethyl sulfoxide (DMSO) was provided by Tianjin Damao Chemical Reagent Factory (Tianjin, China). The water used in this study was purchased from Guangzhou Watson’s Food & Beverage Co., Ltd. (Guangzhou, China). GA, PUN, COR, TGG, CHG, CHI, and EA, all with purities above 98%, were obtained from Shanghai Yuanye Biotechnology Co., Ltd. (Shanghai, China). CA, 4GS, 3GS, CHN, and UM5 were isolated and prepared in our laboratory, and their purity was verified to be greater than 98% by UPLC-UV. The structures of the focused compounds are displayed in Supplementary Figure S9.

Next, 75 batches of samples, including 35 batches of CF (CF1–CF35), 28 batches of CFI (CFI-1–CFI-28), 6 batches of TBF (TBF1–TBF6), 3 batches of PF (PF1–PF3), and 3 batches of CAF (CAF1–CAF3), were purchased from different markets of medicinal materials and authenticated by Professor Xiaoxuan Tian, and were then deposited at the State Key Laboratory of Component-based Chinese Medicine, as detailed in Supplementary Table S1.

Fresh CF samples were collected in October from Yongde County, Lincang City, Yunnan Province, China (altitude: 1051 m, coordinates: 99.44° E and 24.13° N). Fruits weighing 14.02 ± 1.98 g were selected, and five fruits (66.96–73.43 g) were collectively classified as one group. The samples were dried in sunlight (ground temperature: 30.2–43.5 °C) and collected at 0, 2, 4, 8, 16, 20, 24 and 28 days. Then, the weight was recorded and the materials were cut into 2–3 mm slices and dried by a freeze-dryer (FDU-2110, Tokyo Rikakikai Co., Ltd., Tokyo, Japan) until a constant weight was reached. The fresh fruits were peeled into exocarp, mesocarp, and endocarp and seeds, and exposed to sunlight-drying. All of these steps were repeated thrice and the samples were deposited in the State Key Laboratory of Component-based Chinese Medicine, Tianjin University of Traditional Chinese Medicine.

### 3.2. Preparation of Standard and Sample Solution

#### 3.2.1. Preparation of Standard Solution

Twelve standard compounds were weighed precisely. Then, CA and GA were dissolved in water; 4GS, 3GS, COR, and TGG were dissolved in methanol; and PUN, CHN, UM5, CHI, CHG, and EA were dissolved in dimethyl sulfoxide, respectively. These steps were employed to provide a mixed solution with final concentrations of CA (0.501 mg/mL), GA (0.211 mg/mL), 4GS (0.051 mg/mL), 3GS (0.066 mg/mL), PUN (0.243 mg/mL), COR (0.066 mg/mL), TGG (0.068 mg/mL), CHN (0.231 mg/mL), UM5 (0.036 mg/mL), CHI (0.184 mg/mL), CHG (0.186 mg/mL), and EA (0.175 mg/mL). Subsequently, a series of diluted mixed standard solutions were used to construct the calibration curves.

#### 3.2.2. Sample Preparation

The whole CF fruit samples were smashed to remove the endocarp and seeds, then pulverized. The CFI, TBF, PF, and CAF samples were pulverized directly. In view of the properties of hydrolyzable tannins, the factors that could affect the extraction outcome were investigated and optimized, including the extraction solvent (20%, 40%, 60%, 80% methanol aqueous solution, and methanol), solid-liquid ratio (1:50, 1:125, 1:250, and 1:500), ultrasonic time (10, 20, and 30 min), and ultrasonic temperature (30, 40, and 50 °C). The optimal sample extraction conditions were as follows: Sample powder (0.2 g) was transferred into a 50 mL volumetric flask and then ultrasonically extracted by a sonicator (600 W, DL-720B, Shanghai Zhixin Instrument Co., Ltd., Shanghai, China) using methanol with ultrasonic power for 20 min. After cooling down to room temperature, the extracted sample solution was diluted to scale by adding methanol and centrifuged at 13,700 rpm for 10 min to afford the supernatant before analysis.

### 3.3. UPLC-PDA Analysis

ACQUITY UPLC H-class plus system (Waters Corporation, Milford, MA, USA) was used to perform chromatographic separation with a COSMOSIL PBr column (2.1 mm × 100 mm, 2.6 μm, Nacalai Tesque, Inc., Kyoto, Japan) at 30 °C. The mobile phase was composed of 0.1% formic acid aqueous solution (A) and methanol (B) and implemented in the gradient elution as follows: 0–5 min, 0–5% B; 5–9 min, 5–30% B; 9–12 min, 30–33% B; 12–19 min, 33–50% B; 19–28 min, 50–81% B; 28–30 min, 81–100% B; and 30–33 min, 100% B. The flow rate was 0.3 mL/min and the injection volume was 2 μL. The detection wavelength was set at 270 nm.

### 3.4. Methodological Validation

The linearity, limit of detection (LOD), limit of quantification (LOQ), precision (both intra- and inter-day), stability, reproducibility, and recovery were validated to assess the applicability of the analytical method for quantitative analysis of the twelve compounds. The calibration curves were established by plotting the concentration (*x*) against the peak area (*y*) of the tested compounds. The LOD and LOQ were determined by injecting a series of diluted standard solutions with certain concentrations until the signal-to-noise ratios (S/N) reached 3 and 10, respectively. The intra- and inter-day precisions were calculated by analyzing six replicate injections on the same day and on three consecutive days, respectively. To evaluate the stability of the sample solution, repeated injections were performed at 0, 2, 4, 6, 8, 10, and 12 h under room temperature. The repeatability was verified using six prepared samples from the same source. The recovery test was conducted by adding the standard solutions into 0.1 g sample powder, then following the above-described sample preparation procedure, which was executed in parallel for six repetitions.

### 3.5. UHPLC-QTOF-MS Analysis

An ultra-high performance liquid chromatographic system (Agilent 1260 Infinity II) was coupled with a 6550 QTOF™ high-resolution mass spectrometer (Agilent, Santa Clara, CA, USA) in the negative ESI (electrospray ionization) mode. Chromatographic separation was achieved on a COSMOSIL PBr column (2.1 mm × 100 mm, 2.6 μm) at 30 °C. The mobile phase composed of 0.1% formic acid in water (A) and methanol (B) was run at a flow rate of 0.3 mL/min. The injection volume was 5 μL. The elution program was consistent with the above-described UPLC-PDA analysis method. All samples were injected in the negative ion mode. The ion source parameters of the high-resolution QTOF-MS were set as follows: gas temperature, 200 °C; drying gas, 12 L/min; nebulizing pressure, 40 psi; sheath gas temperature, 350 °C; sheath gas flow, 11 L/min; nozzle voltage, 1.0 kV; capillary voltage, −3.5 kV; fragmentor, 390 V; collision energy (CE), 30 eV. The TOF analyzer scanned the mass-to-charge ratio (*m*/*z*) range of 100–1500 for MS^1^ and 50–1500 for MS^2^. The acquisition rates for MS^1^ and MS^2^ were 3 spectra/s and 4 spectra/s, respectively. The precursor ions with the top 3 highest intensities in the MS^1^ spectra were automatically selected to trigger the MS/MS fragmentation by collision-induced dissociation with a threshold of 100 counts.

### 3.6. Data Analysis

The Originpro 2020 SR1 software (Originlab Corp., Northampton, MA, USA), SIMCA 14.1 software (Umetrics, Umea, Sweden), and R4.2.2-programming language were used for the statistical analysis.

## 4. Conclusions

In this study, the common PTCs, including six phenolcarboxylic acids and six HTs, were quantitatively determined by an established UPLC-PDA method, which showed discrimination potential for CF, CFI, TBF, PF, and CAF by HCA and PCA. The abundance of the studied PTCs in the exocarp, mesocarp, and endocarp and seed of CF was further revealed and the HTs (chebulic ellagitannins, gallotannins, and ellagitannins) were observed to be easily degraded to three main phenolcarboxylic acids (CA, GA, and EA) during sunlight-drying of CF. The degradation pathways of representative HTs (CHG, CHI, CHN, and PUN) were finally proposed. Our study sheds light on the quality analysis, selection of medicinal parts, processing, storage for CF, and discrimination of CF from the adulterants, conducing to improving the quality of CF and related products.

## Figures and Tables

**Table 1:** Methodological validation for simultaneous quantification of twelve compounds in CF.

Compounds	Linear Regression			LOD	LOQ	Precision (RSD, %)		Repeatability	Stability
	Regression Equation	*r* ^2^	Linear Range (μg/mL)	(μg/mL)	(μg/mL)	Intra-Day	Inter-Day	(*n* = 6, RSD, %)	(*n* = 7, RSD, %)
CA	*y* = 5584.6*x* + 6891.1	0.9999	7.823–500.7	0.9779	1.956	0.80	0.80	0.7	0.9
GA	*y* = 23,460*x* + 20342	0.9999	3.294–210.8	0.4118	0.8236	1.00	0.40	1.1	0.8
4GS	*y* = 12,053*x* + 2204.6	0.9999	0.7984–51.10	0.0998	0.3992	0.80	0.40	1.2	0.7
3GS	*y* = 13,392*x* + 4111.8	0.9999	1.039–66.48	0.1298	0.5194	1.00	0.40	2.9	1.0
^#^ PUN	*y* = 15,351*x* + 8207.5	0.9999	3.795–242.9	0.4744	0.9488	0.60	0.70	1.4	1.2
COR	*y* = 13,855*x* + 3831.5	0.9999	1.024–65.52	0.1228	0.2559	1.70	0.90	1.4	1.2
TGG	*y* = 19,369*x* + 6044.1	0.9998	1.068–68.32	0.1334	0.2669	2.70	0.60	3.0	0.6
CHI	*y* = 9103.7*x* + 7343.6	0.9999	3.606–230.8	0.4507	0.9014	0.50	0.30	1.4	0.5
UM5	*y* = 32,378*x* + 3274.4	0.9999	0.5578–35.70	0.1395	0.2789	1.00	1.00	1.2	0.9
CHG	*y* = 12,668*x* + 8627.8	0.9999	2.869–183.6	0.3586	0.7172	0.50	0.60	0.7	0.2
CHN	*y* = 13,309*x* + 10,763	0.9999	2.913–186.4	0.3641	0.7281	1.70	0.70	2.7	0.3
EA	*y* = 35,524*x* + 16,314	0.9999	2.739–175.3	0.3424	0.6848	2.40	0.70	0.9	0.9

^#^: the content of PUN is the total content of PUN A and PUN B.

## Data Availability

Data are contained within the article and Supplementary Materials.

## Supplementary Materials

The following supporting information can be downloaded at https://www.mdpi.com/article/10.3390/molecules29102399/s1, Figure S1: The box plot of contents of the observed 12 compounds in the tested samples; Figure S2: Outliers identified from CF (A), CFI (B), TBF (C), PF (D), and CAF (E) based on PCA scores; Figure S3: Discrimination of CF, CFI, TBF, PF, and CAF samples by PCA; Figure S4: Variable importance in projection (VIP) of the tested compounds (A). The results of flesh (B) and whole fruit (C) samples permutations test in the OPLS-DA. (*n* = 200); Figure S5: The cleavage law of products a1–a3 from CHG; Figure S6: The cleavage law of products b2 (A), b1 (B), and b3–b6 (C) from CHI; Figure S7: The cleavage law of products c1–c2 from CHN; Figure S8: The cleavage law of products d1–d2 from PUN; Figure S9: The structures of six hydrolyzable tannins and six phenolcarboxylic acids in CF; Table S1: The detailed information of sample collected from different origins.

## Abbreviations

CA	Chebulic acid
CAF	Canarii Fructus
CF	Chebulae Fructus
CFI	Chebulae Fructus Immaturus
CHG	Chebulagic acid
CHI	Chebulanin acid
CHN	Chebulinic acid
COR	Corilagin
EA	Ellagic acid
GA	Gallic acid
HCA	Hierarchical cluster analysis
HTs	Hydrolyzable tannins
PCA	Principal component analysis
PF	Phyllanthi Fructus
PUN	Punicalagin
PTCs	Phenolcarboxylic and tannic constituents
TBF	Terminaliae Belliricae Fructus
TGG	1,3,6-tri-*O*-galloyl-β-d-glucose
UPLC-PDA	Ultra-performance liquid chromatography with a photo-diode array detector
UM5	Urolithin M5
3GS	3-*O*-galloyl-(−)-shikimic acid
4GS	4-*O*-galloyl-(−)-shikimic acid
