# Supplementary material for: Study on Quality Characteristic of Chebulae Fructus and Its Adulterants and Degradation Pathway of Hydrolyzable Tannins
Source: Molecules. 2024 May 20;29(10):2399. doi: 10.3390/molecules29102399 (PMC11123712; doi:10.3390/molecules29102399)
Supplement: Supplementary file 1 [file molecules-29-02399-s001.zip › molecules-2940490-supplementary.pdf]

Supplementary Materials

Study on Quality Characteristic of Chebulae Fructus and Its Adulterants and Degradation Pathway of Hydrolyzable Tannins

Jian Xu [1,†], Xiangdong Wang [1,†], Huijuan Yu [1,2], Xin Chai [1,2], Min Zhang [1,2,*], Hong-Hua Wu [1,2,*] and Yuefei Wang [1,2,*]

[1] National Key Laboratory of Chinese Medicine Modernization, State Key Laboratory of Component-Based Chinese Medicine, Tianjin Key Laboratory of TCM Chemistry and Analysis, Tianjin University of Traditional Chinese Medicine, Tianjin 301617, China; xjwyq123456@163.com (J.X.); xiangdongblue@163.com (X.W.); huijuanyu@tjutcm.edu.cn (H.Y.); chaix0622@tjutcm.edu.cn (X.C.)

[2] Haihe Laboratory of Modern Chinese Medicine, Tianjin 301617, China

* Correspondence: zhangm036@tjutcm.edu.cn (M.Z.); wuhonghua2011@tjutcm.edu.cn (H.-H.W.); wangyf0622@tjutcm.edu.cn (Y.W.); Tel.: +86-22-59596366 (M.Z. & H.-H.W. & Y.W.)

† These authors contributed equally to this work.

Table of Contents

Figure S1: The box plot of contents of the observed 12 compounds in the tested samples

Figure S2: Outliers identified from CF (A), CFI (B), TBF (C), PF (D), and CAF (E) based on PCA scores.

Figure S3: Discrimination of CF, CFI, TBF, PF, and CAF samples by PCA.

Figure S4: Variable importance in projection (VIP) of the tested compounds (A). The results of flesh (B) and whole fruit (C) samples permutations test in the OPLS-DA. (n = 200)

Figure S5: The cleavage law of products a1 – a3 from CHG.

Figure S6: The cleavage law of products b2 (A), b1 (B), and b3 – b6 (C) from CHI.

Figure S7: The cleavage law of products c1 – c2 from CHN.

Figure S8: The cleavage law of products d1 – d2 from PUN.

Figure S9: The structures of six hydrolyzable tannins and six phenolcarboxylic acids in CF.

Table S1: The detailed information of sample collected from different origins.

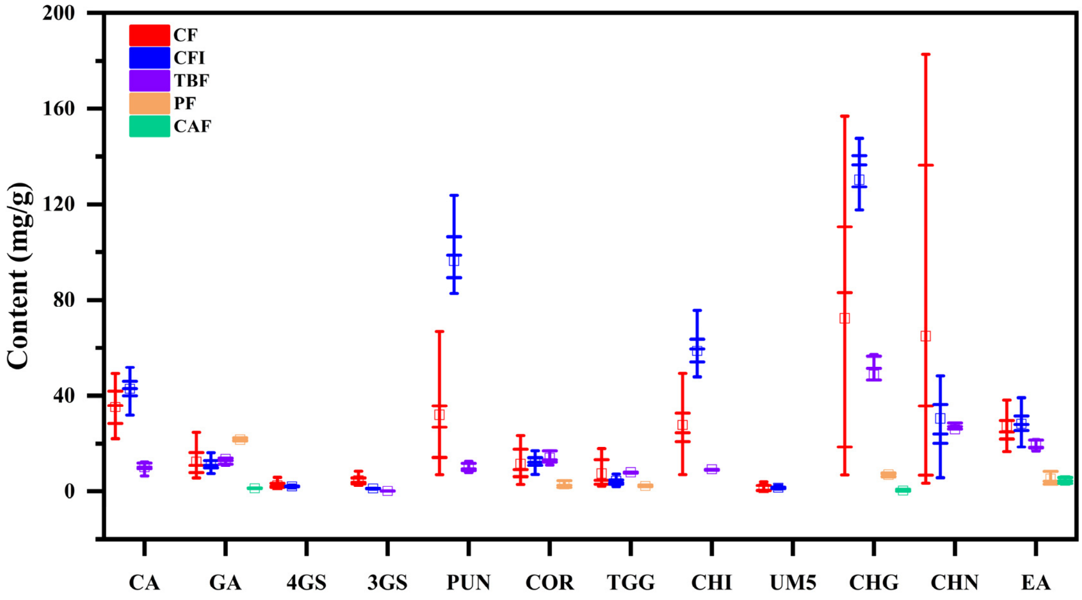

Figure S1. The box plot of contents of the observed 12 compounds in the tested samples

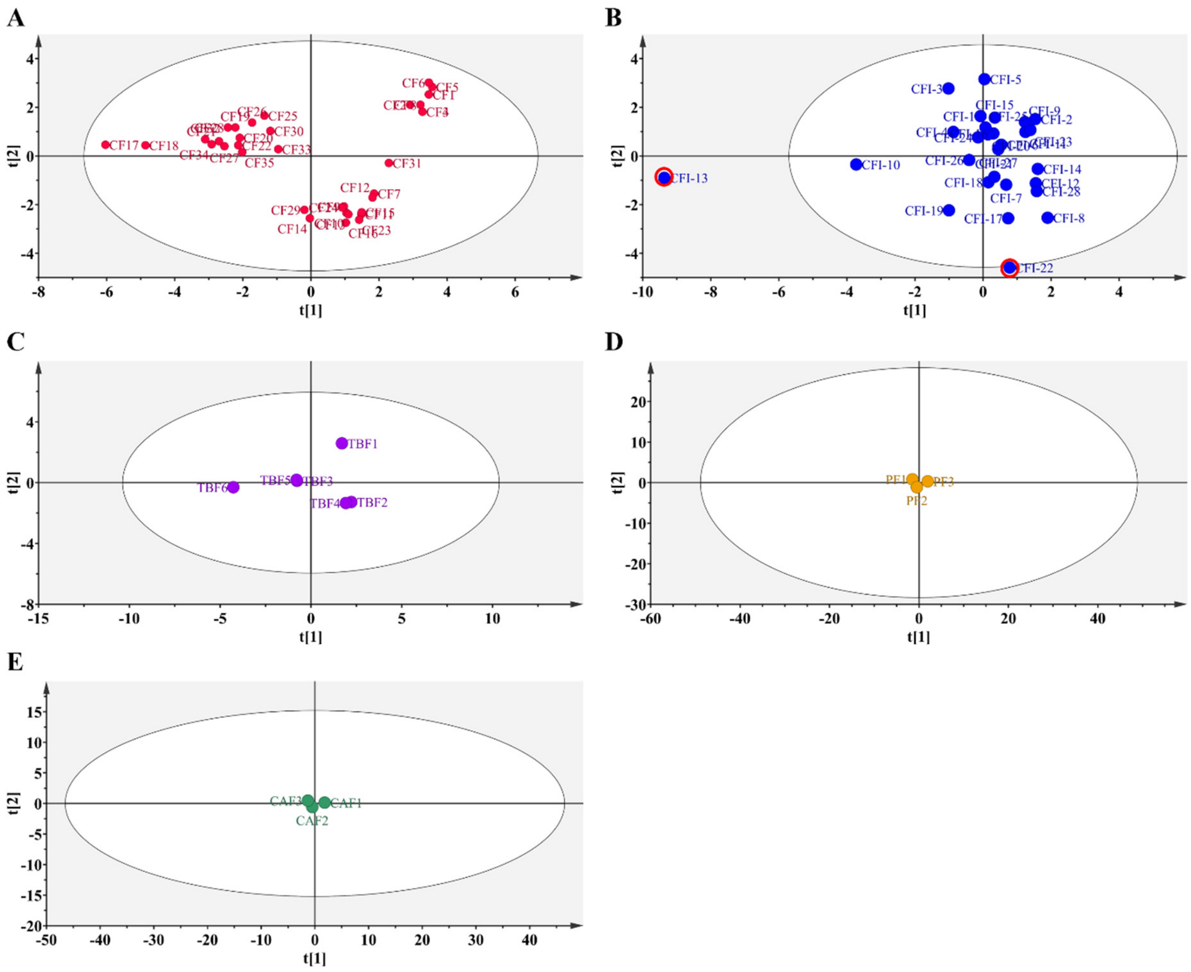

Figure S2. Outliers identified from CF (A), CFI (B), TBF (C), PF (D), and CAF (E) based on PCA scores.

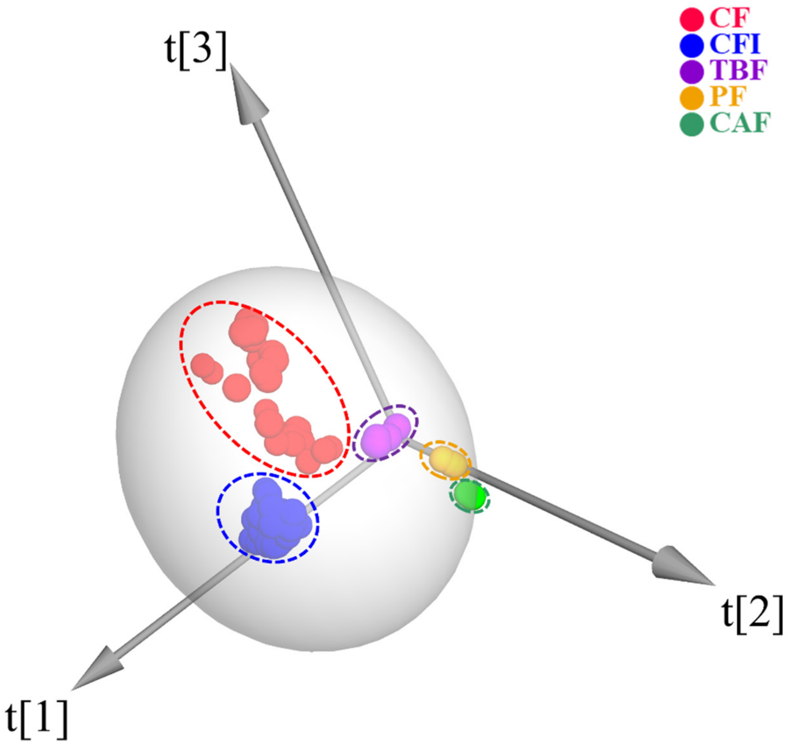

Figure S3. Discrimination of CF, CFI, TBF, PF, and CAF samples by PCA.

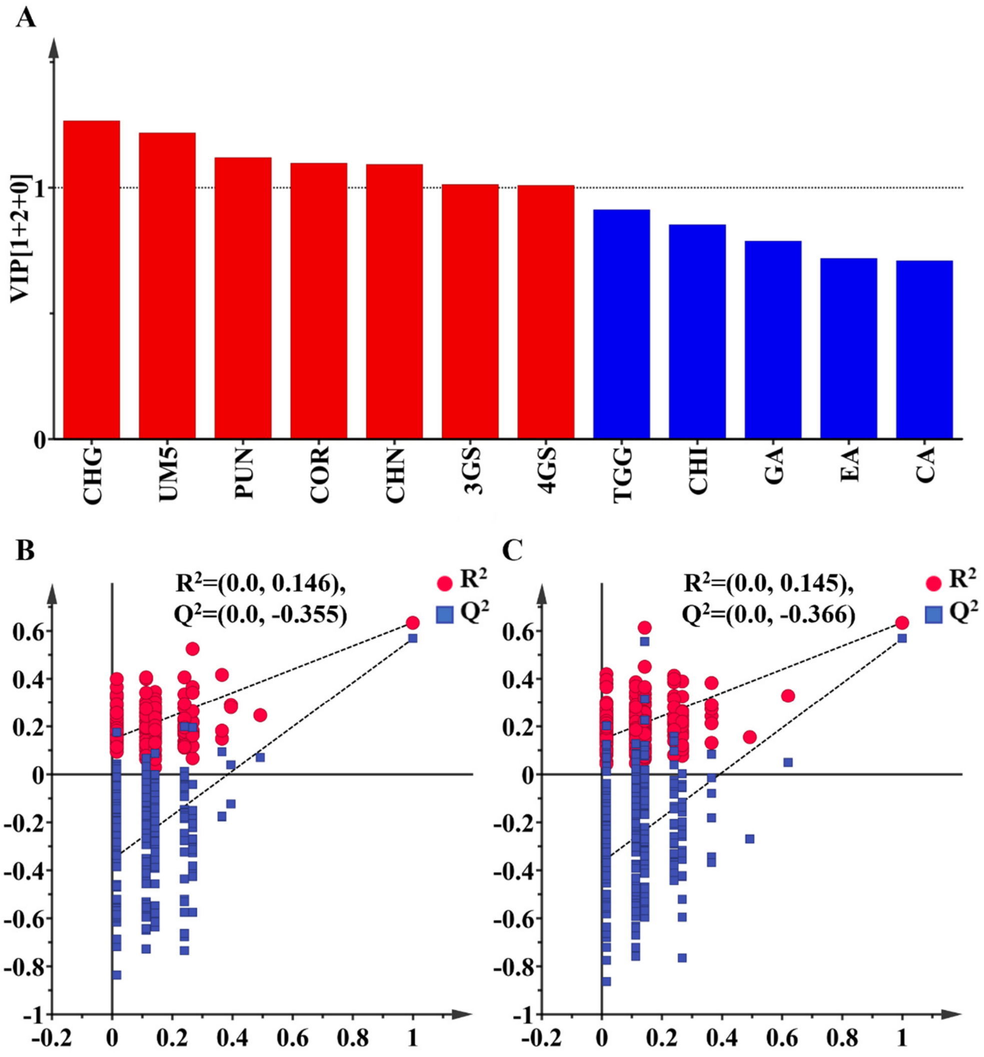

Figure S4. Variable importance in projection (VIP) of the tested compounds (A). The results of flesh (B) and whole fruit (C) samples permutations test in the OPLS-DA. (n = 200)

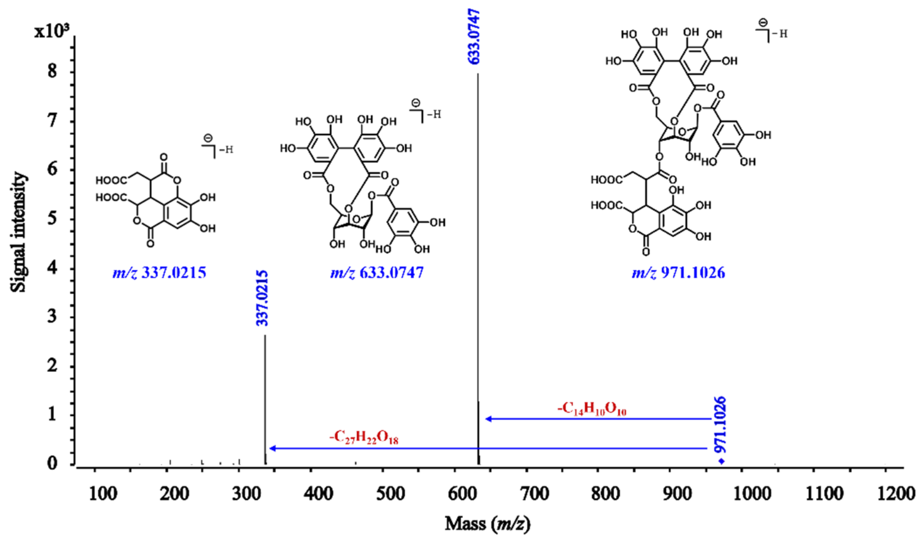

Figure S5. The cleavage law of products a1 – a3 from CHG.

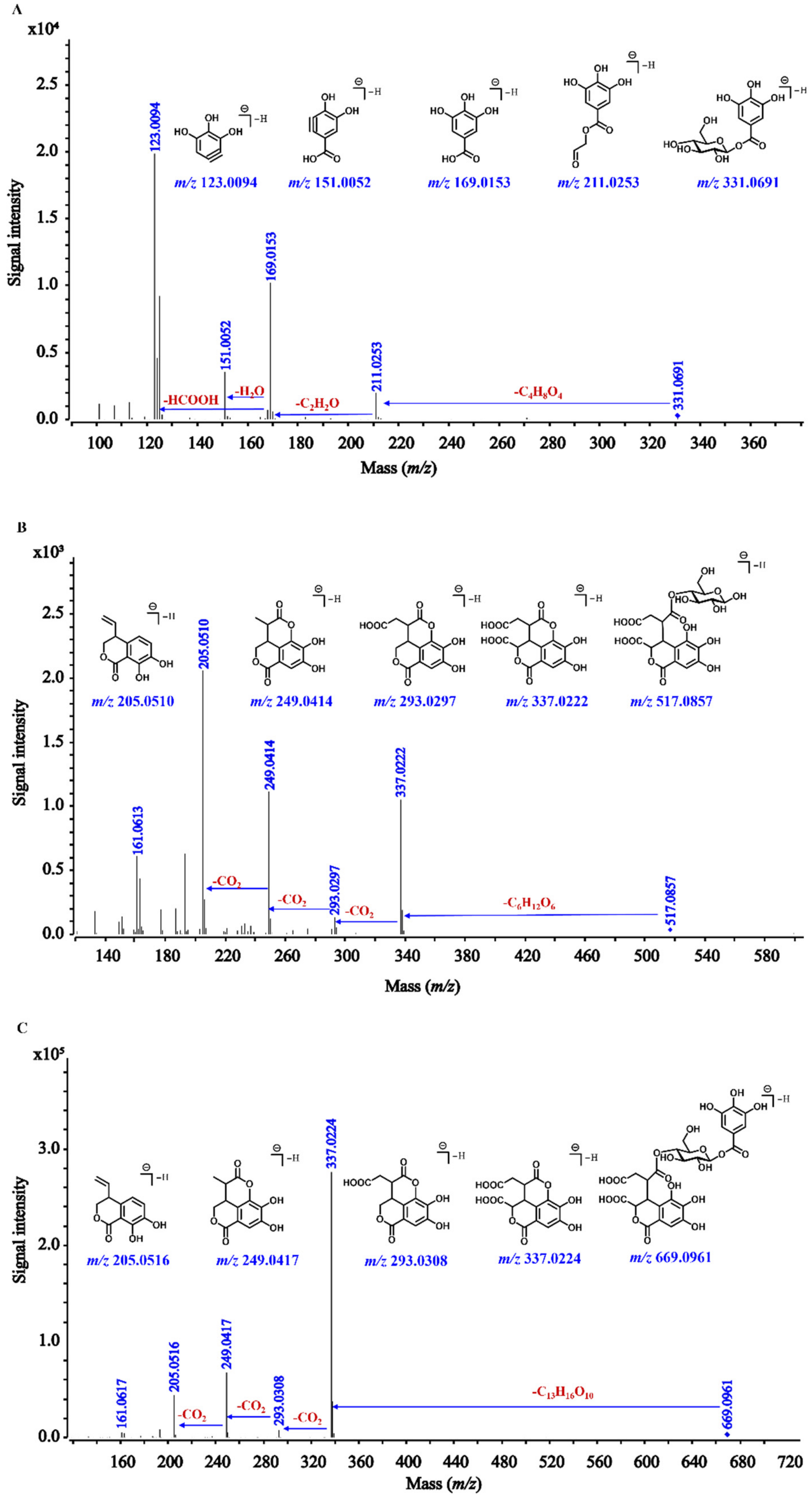

Figure S6. The cleavage law of products b2 (A), b1 (B), and b3 – b6 (C) from CHI.

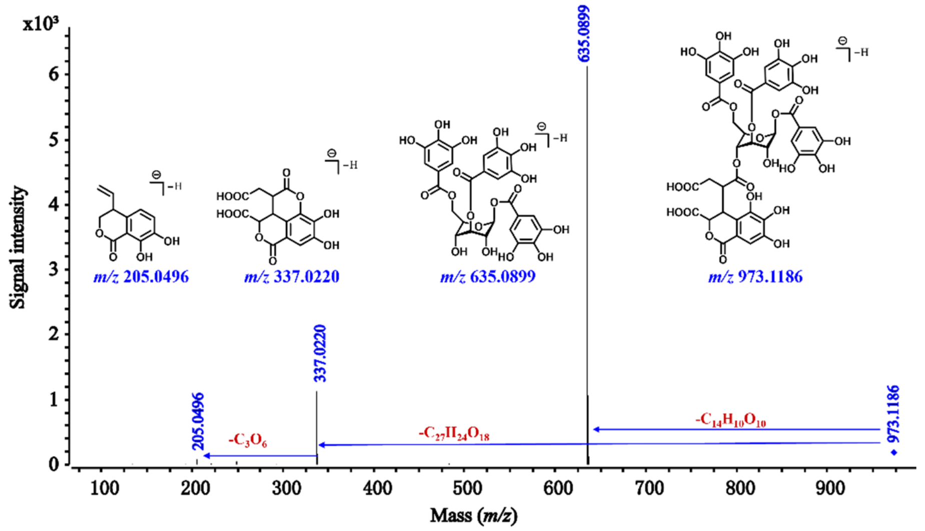

Figure S7. The cleavage law of products c1 – c2 from CHN.

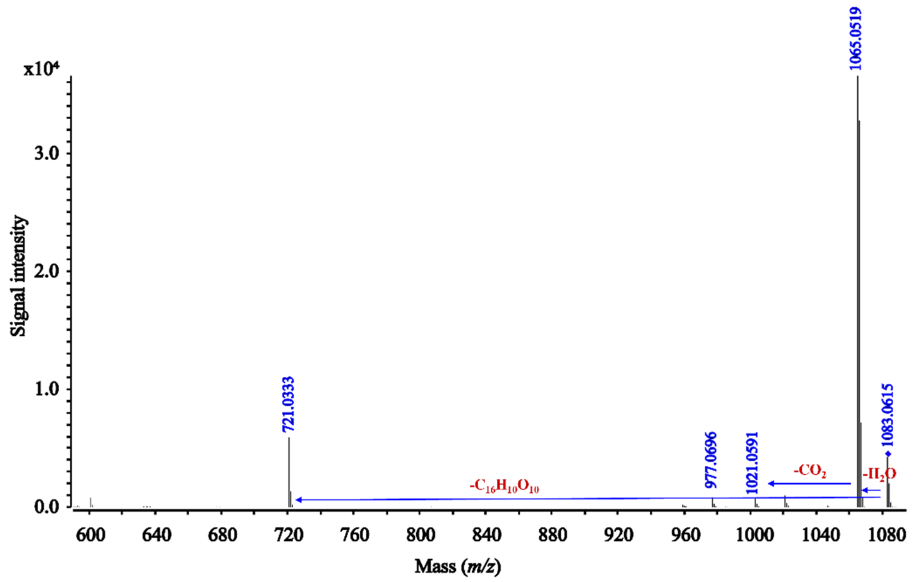

Figure S8. The cleavage law of products d1 – d2 from PUN

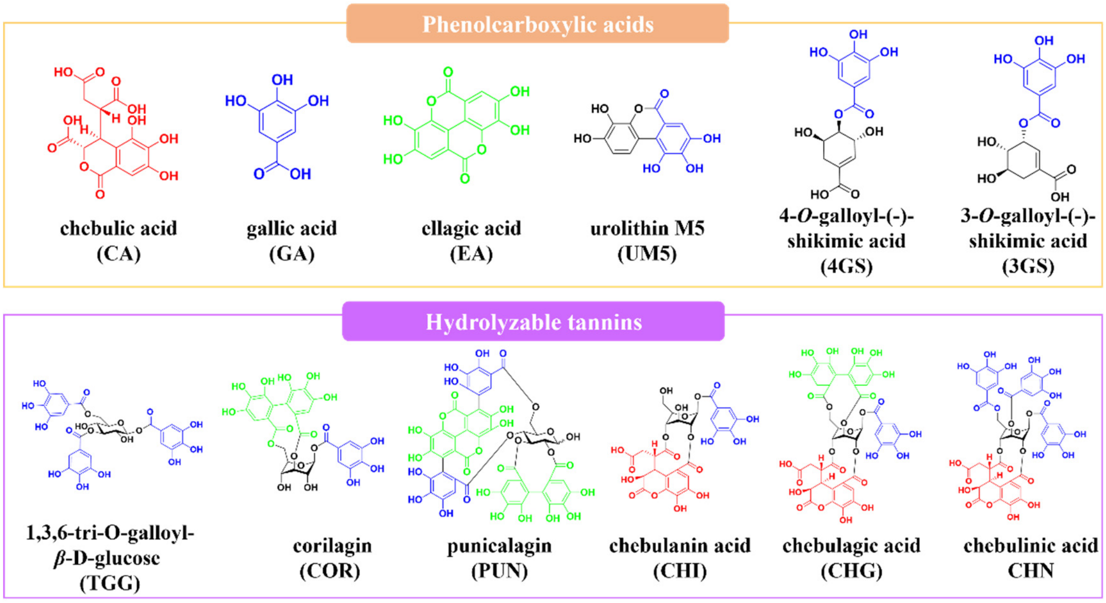

Figure S9. The structures of six hydrolyzable tannins and six phenolcarboxylic acids in CF.

Table S1. The detailed information of samples collected from different origins

Batch No.	Origin	Provider	WF or FF#
CF1	Indian	Tianjin Institute for Drug Control, China	WF
CF2	Indian	Tianjin Institute for Drug Control, China	WF
CF3	Indian	Tianjin Institute for Drug Control, China	WF
CF4	Indian	Tianjin Institute for Drug Control, China	WF
CF5	Indian	Tianjin Institute for Drug Control, China	WF
CF6	Indian	Tianjin Institute for Drug Control, China	WF
CF7	Indian	Tianjin Institute for Drug Control, China	WF
CF8	Indian	Tianjin Institute for Drug Control, China	WF
CF9	Qinghai, China	Chinese Herbs Market in Bozhou, China	WF
CF10	Qinghai, China	Chinese Herbs Market in Bozhou, China	WF
CF11	Yunnan, China	Chinese Herbs Market in Bozhou, China	WF
CF12	Yunnan, China	Chinese Herbs Market in Bozhou, China	WF
CF13	Guangxi, China	Chinese Herbs Market in Bozhou, China	WF
CF14	Yunnan, China	Chengdu Hehuachi Chinese Herbal Mdeicine Market, China	WF
CF15	Yunnan, China	Chengdu Hehuachi Chinese Herbal Mdeicine Market, China	WF

CF16	Xizang, China	Chengdu Hehuachi Chinese Herbal Mdeicine Market, China	WF
CF17	Myanmar	Anguo Traditional Chinese Medicine Digital Palace, China	WF
CF18	Myanmar	Anguo Traditional Chinese Medicine Digital Palace, China	WF
CF19	Myanmar	Anguo Traditional Chinese Medicine Digital Palace, China	WF
CF20	Myanmar	Anguo Traditional Chinese Medicine Digital Palace, China	FF
CF21	Yunnan, China	Beijing Tongrentang Tianjin Hexi Pharmacy Co., Ltd., China	FF
CF22	Yunnan, China	Beijing Tongrentang Tianjin Hexi Pharmacy Co., Ltd., China	FF
CF23	India	Anguo Traditional Chinese Medicine Digital Palace, China	WF
CF24	Yunnan, China	Guangdong Huiqun Traditional Chinese Medicine Co., Ltd., China	WF
CF25	Yunnan, China	Anguo Rendexing Medicinal Materials Co., Ltd., China	FF
CF26	Yunnan, China	Anguo Rendexing Medicinal Materials Co., Ltd., China	WF
CF27	Guangxi, China	Hebei Chunkai Pharmaceutical Co., Ltd., China	FF
CF28	Guangxi, China	Hebei Chunkai Pharmaceutical Co., Ltd., China	WF
CF29	Yunnan, China	Bozhou Zexintang Pharmaceutical Co., Ltd., China	WF
CF30	Guangxi, China	Beijing Boaitang Chinese Medicine Technology Co., Ltd., China	FF
CF31	Guangdong,	Kangmei Pharmaceutical Co., Ltd., China	FF
CF32	Xizang,	Hebei Niuentang E-commerce Co., Ltd., China	WF

CF33	Guangxi, China	Jiuzhouhengyuan Anguo Pharmaceutical Co., Ltd., China	FF
CF34	Guangxi, China	Hebei Huadu Pharmaceutical Co., Ltd., China	FF
CF35	—	Chinese Institute for Food and Drug Control, China	FF
CFI-1	India	Tianjin Institute For Drug Control, China	WF
CFI-2	India	Tianjin Institute for Drug Control, China	WF
CFI-3	India	Tianjin Institute for Drug Control, China	WF
CFI-4	India	Tianjin Institute for Drug Control, China	WF
CFI-5	India	Tianjin Institute for Drug Control, China	WF
CFI-6	India	Tianjin Institute for Drug Control, China	WF
CFI-7	Yunnan, China	Chengdu Hehuachi Chinese Herbal Mdeicine Market, China	WF
CFI-8	Xizang, China	Chengdu Hehuachi Chinese Herbal Mdeicine Market, China	WF
CFI-9	Yunnan, China	Chinese Herbs Market in Bozhou, China	WF
CFI-10	Yunnan, China	Chinese Herbs Market in Bozhou, China	WF
CFI-11	Guangxi, China	Chinese Herbs Market in Bozhou, China	WF
CFI-12	Guangxi, China	Chinese Herbs Market in Bozhou, China	WF
CFI-13	Guangxi, China	Beijing Boaitang Anguo Chinese Medicine Technology Co., Ltd., China	WF
CFI-14	Guangxi, China	Anguo Rendexing Medicinal Materials Co., Ltd., China	WF

CFI-15	Guangxi, China	Hebei Huadu Pharmaceutical Co., Ltd., China	WF
CFI-16	Xizang, China	Hebei Niuentang E-commerce Co., Ltd., China	WF
CFI-17	Xizang, China	Bozhou Zexintang Pharmaceutical Co., Ltd., China	WF
CFI-18	Guangdong, China	Jiuzhouhengyuan Anguo Pharmaceutical Co., Ltd., China	WF
CFI-19	Sichuan, China	Hebei Chunkai Pharmaceutical Co., Ltd., China	WF
CFI-20	Yunnan, China	Beijing Tongrentang Tianjin Hexi Pharmacy Co., Ltd., China	WF
CFI-21	Yunnan, China	Kangmei Pharmaceutical Co., Ltd., China	WF
CFI-22	Qinghai, China	Chinese Herbs Market in Bozhou, China	WF
CFI-23	Hainan	Zequn Chinese Traditional Chinese Medicine Co., Ltd., China	WF
CFI-24	—	Chinese Institute for Food and Drug Control, China	WF
CFI-25	Myanmar	Anguo Traditional Chinese Medicine Digital Palace, China	WF
CFI-26	Myanmar	Anguo Traditional Chinese Medicine Digital Palace, China	WF
CFI-27	Myanmar	Anguo Traditional Chinese Medicine Digital Palace, China	WF
CFI-28	Yunnan, China	Anguo Traditional Chinese Medicine Digital Palace, China	WF
TBF1	Yunnan, China	Chinese Herbs Market in Bozhou, China	WF
TBF2	Qinghai, China	Chinese Herbs Market in Bozhou, China	WF
TBF3	Qinghai, China	Chinese Herbs Market in Bozhou, China	WF

TBF4	Xinjiang, China	Chinese Herbs Market in Bozhou, China	WF
TBF5	Xinjiang, China	Chinese Herbs Market in Bozhou, China	WF
TBF6	Xinjiang, China	Chinese Herbs Market in Bozhou, China	WF
PF1	India	Taobao.com, China	WF
PF2	Yunnan, China	Taobao.com, China	WF
PF3	Jiangxi, China	Taobao.com, China	WF
CAF1	Fujian, China	Chinese Herbs Market in Bozhou, China	WF
CAF2	Fujian, China	Chinese Herbs Market in Bozhou, China	WF
CAF3	Guangdong, China	Beijing Tongrentang Tianjin Hexi Pharmacy Co., Ltd., China	WF

#: the whole fruit (WF), the flesh of fruit (FF). CF (Chebulae Fructus), CFI (Chebulae Fructus Immaturus), TBF (Terminaliae Belliricae Fructus), PF (Phyllanthi Fructus), and CAF (Canarii Fructus).
